# Pyrosequencing-Based Comparative Genome Analysis of *Vibrio vulnificus* Environmental Isolates

**DOI:** 10.1371/journal.pone.0037553

**Published:** 2012-05-25

**Authors:** Shatavia S. Morrison, Tiffany Williams, Aurora Cain, Brett Froelich, Casey Taylor, Craig Baker-Austin, David Verner-Jeffreys, Rachel Hartnell, James D. Oliver, Cynthia J. Gibas

**Affiliations:** 1 Department of Bioinformatics and Genomics, the University of North Carolina at Charlotte, Charlotte, North Carolina, United States of America; 2 Department of Biology, the University of North Carolina at Charlotte, Charlotte, North Carolina, United States of America; 3 Centre for Environment, Fisheries, and Aquaculture Science, Weymouth, Dorset, United Kingdom; Institute for Genome Sciences, University of Maryland School of Medicine, United States of America

## Abstract

Between 1996 and 2006, the US Centers for Disease Control reported that the only category of food-borne infections increasing in frequency were those caused by members of the genus *Vibrio*. The Gram-negative bacterium *Vibrio vulnificus* is a ubiquitous inhabitant of estuarine waters, and is the number one cause of seafood-related deaths in the US. Many *V. vulnificus* isolates have been studied, and it has been shown that two genetically distinct subtypes, distinguished by 16S rDNA and other gene polymorphisms, are associated predominantly with either environmental or clinical isolation. While local genetic differences between the subtypes have been probed, only the genomes of clinical isolates have so far been completely sequenced. In order to better understand *V. vulnificus* as an agent of disease and to identify the molecular components of its virulence mechanisms, we have completed whole genome shotgun sequencing of three diverse environmental genotypes using a pyrosequencing approach. *V. vulnificus* strain JY1305 was sequenced to a depth of 33×, and strains E64MW and JY1701 were sequenced to lesser depth, covering approximately 99.9% of each genome. We have performed a comparative analysis of these sequences against the previously published sequences of three *V. vulnificus* clinical isolates. We find that the genome of *V. vulnificus* is dynamic, with 1.27% of genes in the C-genotype genomes not found in the E- genotype genomes. We identified key genes that differentiate between the genomes of the clinical and environmental genotypes. 167 genes were found to be specifically associated with environmental genotypes and 278 genes with clinical genotypes. Genes specific to the clinical strains include components of sialic acid catabolism, mannitol fermentation, and a component of a Type IV secretory pathway *VirB4*, as well as several other genes with potential significance for human virulence. Genes specific to environmental strains included several that may have implications for the balance between self-preservation under stress and nutritional competence.

## Introduction

Of all seafood-associated pathogens, none are as critical as those of the genus *Vibrio*, and of all food-borne pathogens, the US Centers for Disease Control reported that only infections by those of this genus increased (by 78%) between 1996 and 2006 [1]. In the United States, 95% of all deaths resulting from seafood consumption are caused by a single bacterium, *Vibrio vulnificu*s [1]. *V. vulnificus* is part of the normal bacterial flora of estuarine waters and occurs in high numbers in molluscan shellfish around the world [2]. In the 10-year period between 2000 and 2009, 303 cases involving oyster ingestion occurred in the United States, of which 148 were fatal (Oliver, unpublished). A COVIS dataset suggest that there were over 1800 *V. vulnificus* cases reported in the USA from 1988–2010, with over 500 associated fatalities (Baker-Austin et al., unpublished). Infections occur rapidly, with median incubation times to onset of symptoms being as little as 7 hours [1]. Most (∼85%) cases occur in males, because females are protected to some extent, from the *V. vulnificus* endotoxin by estrogen [3]. Nearly all infections (∼95%) occur in individuals who are immunocompromised, have diabetes, or who have underlying diseases or syndromes that result in elevated serum iron levels, primarily liver cirrhosis secondary to alcohol abuse/alcoholism [4]. These relatively common conditions put a large number of persons at risk for serious injury or death from *V. vulnificus*, and we would expect to see a far greater number of cases than are typically reported each year. The question then arises as to why so few of these infections are reported each year in the USA.

Understanding the mechanism of *V. vulnificus* virulence and the molecular basis of its interaction with human and oyster hosts is the key to this question. Despite a high degree of phenotypic and genotypic heterogeneity among *V. vulnificus* strains all known putative virulence determinants have been found to be expressed in both clinical and environmental isolates [1]. Despite this, Starks et al. (2000) [5] found clinical isolates (n = 3) to be significantly more virulent than environmental strains (n = 3) in both an intraperitoneal and subcutaneous mouse model, and we have found 81% of 16 C-genotype strains examined to be virulent (LD_50_ ≤10^3^), but only 31% of 13 E-genotype strains in an iron-overload mouse model (Oliver, unpublished).

Several approaches have been used to identify genotypic factors that distinguish between virulent and avirulent isolates of this pathogen. Aznar et al. [6] identified two groups (termed A and B) of *V. vulnificus* strains based on 16S rDNA gene polymorphism, and Nilsson et al. [7] showed that these two groups were associated with clinical (B) or environmental (A) isolation. Despite employing a variety of population genetics methods, however, Gutacker et al. [8] found no association between their grouping and environmental or clinical origin. Recently, Okura et al. [9] employed a PCR-based assay, using a primer pair derived from a group-specific sequence of a RAPD-PCR fragment encoding a hypothetical protein, to distinguish pandemic strains of *V. parahaemolyticus* from non-pandemic strains. Using the same strategy, we identified an approximately 200 bp RAPD-PCR amplicon significantly associated with clinical isolates [10]. Analysis of this *vcg* (termed the Virulence Correlated Gene) led to a PCR-based assay that can separate *V. vulnificus* into two groups which strongly correlate to the source (clinical or environmental) of their isolation [11]. In a subsequent study of the distribution of the C- and E-genotypes in oysters and the surrounding estuarine waters, we found that while an almost equal distribution of the two genotypes existed in water, the E-genotype accounted for over 84% of those present in oysters [12]. This suggests that either E-genotypes are preferentially taken up by oysters, or that they survive better than do C-genotypes following uptake. More recently, Baker-Austin et al. [13] developed a rapid, real-time PCR method for *in situ* detection of C-genotype *V. vulnificus* strains present in raw oysters. These two genotypes may in fact be different ecotypes, as the genetic dimorphisms are not limited to the *vcg* gene, but occur throughout the chromosome and appear to dictate the species' environmental preference [11], [14]. Despite the growing recognition of the existence of these two genotypes and their relevance to human disease, only clinical strains of the C-genotype have been completely sequenced to date [15], [16], [17]. Recently, a comparative genomic analysis study using short read data was performed on four *V. vulnificus* strains, including three E-strains and ATCC 33149 [18]. However, that study employed ABI SoLID sequencing to produce very short fragment reads. Such reads cannot be assembled *ab initio*, but must be mapped to the C reference genomes. This approach left the possibility that regions of the E genome for which there is no reference in the C sequence remained undetected. In the present study, we report on the sequencing of three strains of the E-genotype of *V. vulnificus*, using Roche 454 GS Titanium sequencing. Genomes have been assembled *ab initio* into large contigs, and the genomic sequences are estimated to be over 99% complete. These newly sequenced genomes have been compared to three previously published C-genotype genomes, strains CMCP6, YJ016, and MO6-24/O. The results of our comparison indicate several significant differences in gene content between the C- and E-genotypes of this pathogen, including genomic regions unique to the E-genotypes, which provide initial insights into the functional basis of pathogenicity in *V. vulnificus*.

## Results and Discussion

### Genome Sequencing and Assembly

**Figure 1:**
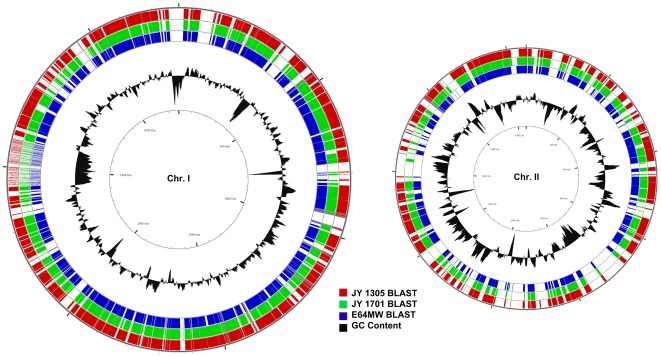
Circular maps of the sequence contigs of *V. vulnificus* JY1305, JY1701, and E64MW. From the outside in, the first circle (red) represents *V. vulnificus* JY1305 genomic contigs, the second circle (green) represents *V. vulnificus* JY1701 genomic contigs, and third circle (blue) represents *V. vulnificus* E64MW genomic contigs. The circles represent BLAST alignment of contigs against the *V. vulnificus* CMCP6 reference genome. Circle 4 shows GC content. Figure generated using CGView [21].

188,710,063 nucleotide bases were generated for *V. vulnificus* strain JY1305. Given the known sizes and expected variability of *V. vulnificus* genomes, we estimated that this is equivalent to ∼33× coverage depth of the *V. vulnificus* JY1305 genome, of estimated size 5.7 Mb. We obtained 671,521 reads of average length 281 bp. The data were assembled into 159 large contigs and 9,184 unassembled fragments using the MIRA assembler, version 3.0 [19]. Table 1 has the complete assembly results for the three E-strain genomes. The coverage of each of these genomes is significantly above the recommended genome coverage (6–10×) for a whole prokaryote genome study established in a recent exhaustive simulation of outcomes of Roche 454 type sequencing in prokaryotes [20]. In Figure 1, we show the assembled contigs from each of the newly sequenced E genomes, aligned to the *V. vulnificus* CMCP6 genome [21]. *V. vulnificus* CMCP6 was recently re-annotated and is regarded as the most complete and accurate of the published *V. vulnificus* clinical strain genomes [22]. Assembled contigs were deposited in the NCBI whole genome shotgun archive, and are available under project IDs 49015 (JY1305), 67135 (E64MW) and 67137 (JY1701). The GenBank accession IDs are AFSW00000000 (JY1305), AFSX00000000 (E64MW), and ASFY00000000 (JY1701) in the NCBI Whole Genome Assembly database. Complete sequence data will be made available via the NCBI Short Read Archive and at http://gibas-research.uncc.edu.

**Table 1:** Summary of assembly and annotation characteristics for the *V. vulnificus* JY1305, E64MW, and JY1701 genomes.

Genomic Characteristic	*V. vulnificus* JY1305	*V. vulnificus* E64MW	*V. vulnificus* JY1701
**# of reads**	671,521	376,287	321,091
**# of nucleotides sequenced**	188,710,063 bp	96,530,017 bp	73,115,338 bp
**Average read length**	281 bp	257 bp	228 bp
**# of contigs**	159	271	329
**N50**	237659 bp	69696 bp	36756 bp
**N90**	54287 bp	14424 bp	9249 bp
**Largest Contig**	489256 bp	163962 bp	112761 bp
**Depth Coverage**	∼33×	∼17×	∼13×
**Estimate Genome Size**	5.7 Mb	5.7 Mb	5.6 Mb
**Genome Coverage**	∼99.9%	∼99%	∼99%
**Chromosome Number**	2	2	2
**Plasmid**	None	N/A	N/A
**G+C content %**	46.7%	46.7%	46.5%
**Predicted Genes**	4235	4301	4425
**# of predicted tRNAs**	115	109	96
**# of predicted rRNAs**	23	17	15

### General properties of the *Vibrio* E strain genomes

The genome of *V. vulnificus* JY1305 is composed of 2 circular chromosomes with an estimated total of approximately 5.7 Mb of genomic DNA. *V. vulnificus* E64MW is estimated to be nearly identical in size, with *V. vulnificus* JY1701 slightly smaller at 5.6 Mb. Some *Vibrio* strains are known to have plasmids, but the *V. vulnificus* JY1305 sequence data contained no evidence of extrachromosomal DNA. PCR validation was performed to verify this finding and no plasmid DNA was found in the genomic DNA preps. It is unknown if *V. vulnificus* E64MW and *V. vulnificus* JY1701 contain plasmid DNA, but no plasmid sequence with homology to known *V. vulnificus* YJ016 plasmid sequences was identified, either in the assembled genomic sequence, or among the unassembled reads.

Table 1 summarizes the general characteristics and predicted gene content of each sequenced draft genome. Complete gene lists for each of the newly sequenced genomes are provided in Supplement S1.

### Locally collinear blocks highlight extensive synteny in the *Vibrio vulnificus* genomes

**Figure 2:**
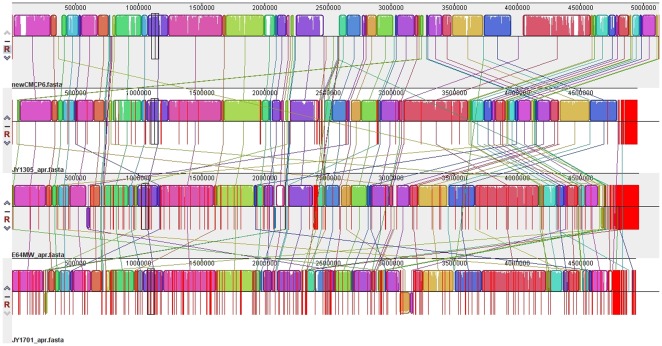
Genomic alignment of *Vibrio vulnificus* Biotype 1 strains CMCP6, YJ016, MO6-24/O, JY1305, E64MW, and JY1701. Locally conserved block based alignment between the reference genome CMCP6 and the newly sequenced genomes of JY1305, E64MW, and JY1701 as locally collinear blocks (LCB). Figure generated using Mauve [23].

LCBs (locally collinear blocks) are defined as conserved segments that appear to be internally free from genome rearrangements relative to the other genomes in the set under study [23]. The newly sequenced *V. vulnificus* strains were co-analyzed with genome sequences of strains CMCP6, YJ016, and MO6-24/O to identify LCBs common to C and E strains. The *V. vulnificus* CMCP6 genome was used as the reference genome in this analysis. At a size threshold of 1% of the genome, or 57 kb or greater, there are a total of 24 locally collinear sequence blocks that are conserved in the six *V.vulnificus* genomes. All of these large blocks are found in each of the six *V. vulnificus* strains, and they cover approximately 68.5% of the genome. At a size threshold of 90 aa (270 bp) or greater, we find an additional 186 LCBs. At this size threshold, LCBs are not necessarily conserved across all six genomes, and may correspond to individual genes or genomic islands that differentiate among the sequenced strains. Figure 2 shows the global arrangement of LCBs identified among the *V. vulnificus* strains (CMCP6, JY1305, E64MW, and JY1701) used in this study. Table S1 contains a table that summarizes the conservation of locally collinear blocks in all 6 *V. vulnificus* genomes, and Supplement S2 contains all LCBs identified, along with their genomic coordinates.

### Genome content comparison

**Figure 3:**
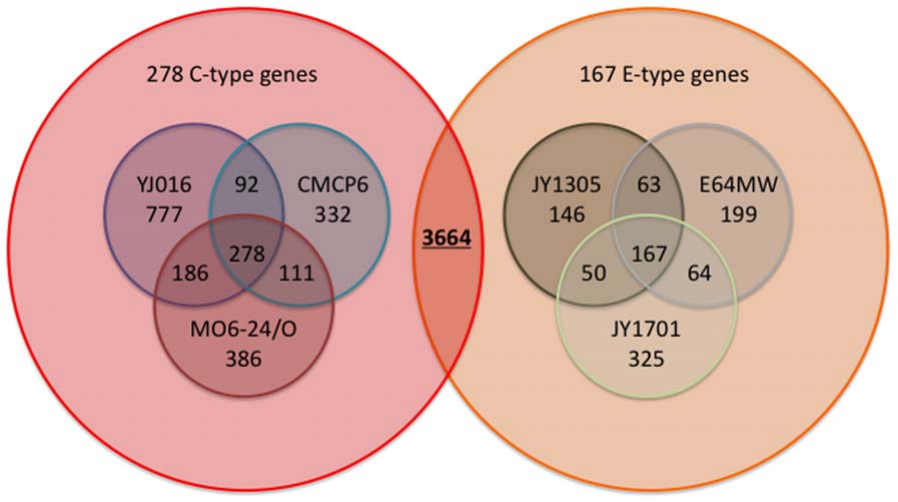
*Vibrio vulnificus* genomic content differential Venn diagram. A 6-way Venn diagram representing the differential and shared gene counts between the *V. vulnificus* YJ016, CMCP6, MO6-24/O, JY1305, E64MW, and JY1701. The main Venn diagram represents the overlap between C- and E- genotype groups, while the nested Venn diagrams represent the content relationships among the individual C-genotype or E-genotype strains. Gene counts are based on presence or absence of orthologs, where orthology is defined by OrthoMCL [74], using as a clustering criterion shared sequence similarity of 70% or greater.

After annotation of the newly sequenced E-genotype *Vibrio vulnificus* genomes as described in Materials and Methods, we performed a comparative analysis of the presence or absence of individual genes. We compared the E-genotype genomes to the group of previously sequenced C-genotype *V. vulnificus* genomes as well as to a broader group of all 16 previously completely sequenced genomes belonging to the genus *Vibrio* (See Materials and Methods). When we subsequently refer to comparisons of E, or C and E types against “all *Vibrio* spp.”, we are referring to this group. Figure 3 summarizes the gene count differentials for the six *V. vulnificus* strains included in this study. Genes were clustered together on the basis of a shared sequence similarity of 70% or greater for the purpose of defining orthology, as described in Materials and Methods. The counts represent differential presence or absence of a gene ortholog in a given genome.

### The conserved core of *V. vulnificus*, and commonalities among *Vibrios*

We identified 1748 core genes that are common to the three draft E-genotype genomes and the 16 *Vibrio* genomes that have been completely characterized at the time of this writing. Each of these genes has a single-copy ortholog in each of the genomes analyzed. 192 genes were identified as core genes to all other *Vibrio* spp. but were not present in any of the *V. vulnificus* genomes, whether C-genotype or E-genotype. 940 genes were identified as core genes found in the six *V. vulnificus* genomes, but were not present in any other *Vibrio* spp. The gene VV2 0404 (*vvhA*), which is commonly used in combination with other markers to distinguish *V. vulnificus* from other *Vibrio* spp. in molecular assays, were found, as expected, in all six *V. vulnificus* strains, which gives us confidence in the sequencing and differential analysis. A related gene, VVA0964, the cytolysin secretion protein gene *vvhB* [24], is unique to the *V. vulnificus* genomes and may have potential as a diagnostic marker. The gene encoding zinc metalloprotease, VV2-0032 (*vvpE*), another commonly-used diagnostic marker, was identified by Gulig et al. 2010 as being common to both E-genotypes and C-genotypes [18], and we found this to be true in our analysis, as well.

Also found in the list of 940 core *V. vulnificus* genes are the Flp pilus genes. We believe this is a novel observation, as we have not seen it discussed elsewhere. The E- and C-genotypes of *V. vulnificus* contain a nearly identical operon for the assembly of an Flp pilus, a type IV pilus that mediates adherence, including genes for Flp pilus assembly *CpaB, CpaC*, a conserved unknown protein, and *CpaE*. The Tad assembly proteins of the Flp pilus, including *TadA*, *TadB*, *TadC*, and *TadD*, are also highly conserved and identically ordered in C7184 and YJ016. Both E- and C- strains of *V. vulnificus* contain all the components of the Tad assembly proteins except *TadD*, while other *Vibrio* spp. do not. These genes may be part of a *tad* (tight adherence) locus, found in a wide variety of bacteria, that is characteristic of horizontal gene transfer. *tad* loci are generally present as part of a mobile genetic element, specifically the “widespread colonization island” [25]. Loci such as these have been shown to be related to diseases, both human and animal, playing a role in colonization and/or pathogenicity. In non-pathogens, *tad* loci are proposed to facilitate environmental niche colonization [26].

Table S2 summarizes key differences between the *V. vulnificus* (Table S2A) and the other *Vibrio* spp (Table S2B). Supplement S3 contains a complete list of all the genes that are differentially present or absent in the *V. vulnificus* C and E strains, relative to all other fully-sequenced *Vibrio* spp.

### Phylogeny of *V. vulnificus* and other *Vibrios*

**Figure 4:**
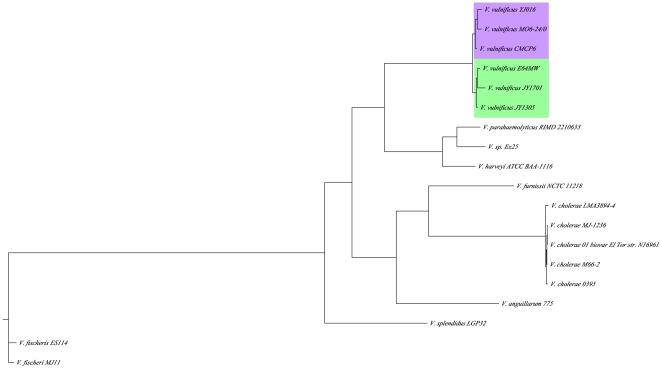
Phylogenetic relationships among sequenced *Vibrio* genomes. Phylogenetic relationships computed using maximum likelihood estimation, from a random sampling of 175 single copy gene ortholog sequences common among the newly sequenced E-genotype genomes and other sequenced *Vibrio* species. Three randomly sampled replicates produce trees with highly similar topologies. Purple box indicates strains classified as C-genotypes and green box indicates strains classified as E-genotypes for *V. vulnificus*.

Figure 4 is a phylogeny of the genus *Vibrio* based on common single copy orthologs. The consensus of the three trees is consistent with the evolutionary relationships previously observed within the genus *Vibrio* [*Vibrio* Phylogeny, PATRIC]. The *V. vulnificus* isolates cluster together, and segregate from the other *Vibrio* spp. Within the *V. vulnificus* clade there is a deep branching between the E-genotypes and C-genotypes and the branchings within the E- and C-genotype groupings are very shallow. This branching suggests a fundamental divergence between the genotypes, which correlates with the divergent lifestyle preferences of E- and C- isolates of *V. vulnificus* [12]. Rosche et al. introduced the concept of distinctive ecotypes in *V. vulnificus* based on eight housekeeping and virulence loci [27] and this distinction is supported by analysis on the genome-wide scale.

### *V. vulnificus* Gene Differentials

We identified an average of 3664 orthologs common to all of the *V. vulnificus* strains analyzed in this study. An in-depth comparison between the two genotypes of *V. vulnificus* revealed 278 genes found only in the C-genotype strains, and 167 genes found only in the E-genotype strains. We also identified 43 genes common to the three C-genotype blood isolates, CMCP6, YJ016, MO6-24/O, and the E-genotype wound isolate, E64MW. Supplement S4 has a complete list of all the genes for these differential categories.

In Table 2 and Table 3, we summarize key differences between C and E genomes, summarizing genes that are shared between the strains of a specific genotype, but excluded from the other genotype. Supplement S4 has a complete list of differentials between the C-genotypes and E-genotypes. A few of those differentiating genes, of significance to human virulence or to survival in the estuarine/oyster environment, are noted here.

**Table 2:** Key differential genes between found in *V. vulnificus* C-genotypes that are NOT present in the E-genotypes.

Strain	Chr.	Locus tag	Product Description	GO id	GO Term
CMCP6	2	VV2_0726	Sialic acid-induced transmembrane protein YjhT	GO:0005975	carbohydrate metabolic process*
	2	VV2_0729	Salic acid utilization regulator RpiR family	GO:0005975	carbohydrate metabolic process*
	2	VV2_0730	N-acetylneuraminate lyase	GO:0008747	N-acetylneuraminate lyase activity*
	2	VV2_0731	TRAP-type transport system large permease component	GO:0016021	Integral to membrane+
	2	VV2_0732	TRAP-type transport system small permease component	N/A	N/A
	2	VV2_0733	TRAP-type system periplasmic component	GO:006810	transport+*
	2	VV2_1509	Putative two –component response regulator & GGDEF family protein YeaJ	GO:0009190	cyclic nucleotide biosynthetic process+*
	2	VV2_1510	Response regulator	GO:0000156	two-component response regulator activity+*
	2	VV2_1106	Arysulfastase A	GO:0008484	sulfuric ester hydrolase activity*
	2	VV2_1107	Arylsulfatase regulator	GO:0008152	metabolic process+*
	2	VV2_1108	Arylsulfatase A	GO:0008449	N-acetylglucosamine-6-sulfatase activity*
	2	VV2_1109	Arysulfatase	GO:0008484	Sulfuric ester hydrolase activity*
	2	VV2_0074	RsbS, negative regulator of sigma-B	N/A	N/A
	2	VV2_0075	anti-sigma B factor RsbT	GO:0005524	ATP binding+
	2	VV2_0076	Serine phosphatase RsbU, regulator of sigma subunit	GO:0008152	metabolic process+*
	2	VV2_0077	Two-component system sensor protein	GO:0004673	protein histidine kinase activity+*
	2	VV2_0735	N-acylmannosamine kinase	GO:0009384	N-acylmannosamine kinase activity*
MO6-24/0	2	VVMO6_03282	Putative two-component response regulator & GGDEF family protein YeaJ	GO:0009190	cyclic nucleotide biosynthetic process+*
	2	VVMO6_03283	Putative two-component response regulator	GO:0003677	DNA binding*
	2	VVMO6_04101	Sialic acid-induced transmembrane protein YjhT	GO:0005975	Carbohydrate metabolic process*
	2	VVMO6_04102	Salic acid utilization regulator RpiR family	GO:0005975	Carbohydrate metabolic process*
	2	VVMO6_04103	N-acetylneuraminate lyase	GO:0008747	N-acetylneuraminate lyase activity*
	2	VVMO6_04104	TRAP-type transport system large permease component	GO:0016021	integral to membrane+
	2	VVMO6_04105	TRAP-type transport system small permease component	N/A	N/A
	2	VVMO6_04106	TRAP-type system periplasmic component	GO:0006810	transport+*
	2	VVMO6_04498	Arysulfastase A	GO:0008484	sulfuric ester hydrolase activity*
	2	VVMO6_04499	GALNS arysulfatase regulator (Fe-S oxidoreductase)	GO:0008152	metabolic process+*
	2	VVMO6_04500	Choline-sulfatase	GO:0008449	N-acetylglucosamine-6-sulfatase activity*
	2	VVMO6_04501	Arysulfastase	GO:0008484	Sulfuric ester hydrolase activity*
	2	VVMO6_03523	rsbS, negative regulator of sigma-B	N/A	N/A
	2	VVMO6_03524	anti-sigma B factor RsbT	GO:0005524	ATP binding+
	2	VVMO6_03525	serine phosphatase RsbU, regulator of sigma subunit	GO:0003824	Catalytic activity+*
	2	VVMO6_03526	two-component system sensor protein	GO:0004673	protein histidine kinase activity+*
	1	VVMO6_02633	PTS system, mannitol-specific IIC component	GO:0016301	kinase activity+*
	1	VVMO6_02634	Mannitol-1-phosphate 5-dehydrogenase	GO:0008926	mannitol-1-phosphate 5-dehydrogenase activity*
	1	VVMO6_02635	Mannitol operon repressor	N/A	N/A
YJ016	2	VVA0202	Transcriptional regulator	GO:0003677	DNA binding*
	2	VVA0325	Putative fimbrial protein Z, transcriptional regulator	GO:0003677	DNA binding*
	2	VVA0326	GGDEF family protein	GO:0009190	cyclic nucleotide biosynthetic process+*
	2	VVA0327	Putative fimbrial protein Z, transcriptional regulator	GO:0003677	DNA binding*
	2	VVA1199	Putative N-acetylneuraminate lyase	GO:0008747	N-acetylneuraminate lyase activity*
	2	VVA1200	TRAP-type C4-dicarboxylate transport system, large permease component	GO:0016021	integral to membrane+*
	2	VVA1201	TRAP-type C4-dicarboxylate transport system, small permease component	N/A	N/A
	2	VVA1202	TRAP-type C4- dicarboxylate transport system, periplasmic component	GO:0006810	Transport+*
	2	VVA1632	Arysulfastase A	GO:0008484	sulfuric ester hydrolase activity*
	2	VVA1633	Arylsulfatase regulator	GO:0055114	oxidation-reduction process+*
	2	VVA1634	Arylsulfatase A	GO:0008449	N-acetylglucosamine-6-sulfatase activity*
	2	VVA1635	Arysulfatase A	GO:0008484	sulfuric ester hydrolase activity*
	2	VVA0581	anti-anti-sigma regulatory factor	N/A	N/A
	2	VVA0582	anti-sigma regulatory factor	GO:000552	ATP binding+
	2	VVA0583	indirect negative regulator of sigma-B activity	GO:0003824	Catalytic activity+*
	2	VVA0584	conserved hypothetical protein	GO:0016310	phosphorylation+*

* indicates there are more than 1 GO term at the lowest level for this gene. +indicates that no significant GO term was associated with gene. Significance adjusted-p value <.005. Box highlights genes that are found on Chromosome 1 of *V. vulnificus* CMCP6. All other differential genes are found on Chromosome 2.

**Table 3:** Key differential genes found in *V. vulnificus* E-genotypes but not in C-genotypes.

Strain	Chr. Alignment	Locus tag	Product Description	GO id	GO Term
**JY1305**	No LCB alignment	VvJY1305_2152	Hypothetical protein	GO:0019627	urea metabolic process*
	LCB in Vv. CMCP6 chr 1	VvJY1305_1632	Permease	GO:0016020	membrane+*
	LCB in Vv. CMCP6 chr 2	VvJY1305_2975	PTS system, glucose-specific IIBBC component	GO:0006810	transport+*
	LCB in Vv. CMCP6 chr 2	VvJY1305_3160	PKD domain containing protein	N/A	N/A
**E64MW**	No LCB alignment	VvE64MW_4158	Hypothetical protein	GO:0016151	nickel ion binding*
	LCB in Vv. CMCP6 chr 1	VvE64MW_1434	Permease	GO:0015128	gluconate transmembrane transporter
	LCB in Vv. CMCP6 chr 2	VvE64MW_3479	PTS system, glucose-specific IIBBC component	GO:0005351	hydrogen symporter activity+*
	No LCB alignment	VvE64MW_3886	PKD domain containing protein	N/A	N/A
**JY1701**	No LCB alignment	VvJY1701_4279	Hypothetical protein	GO:0019627	urea metabolic process*
	LCB in Vv. CMCP6 chr 1	VvJY1701_1508	Permease	GO:0016020	membrane+*
	LCB in Vv. CMCP6 chr 2	VvJY1701_3646	PTS system, glucose-specific IIBBC component	GO:0006810	transport+*
	LCB in Vv. CMCP6 chr 2	VvJY1701_4020	PKD domain containing protein	N/A	N/A

* indicates there are more than 1 GO term at the lowest level for this gene. +indicates that no significant GO term was associated with gene. Significance adjusted-p value <.005. Box highlights differential genes which aligned to locally conserved blocks in Chromosome 1 of *V. vulnificus* CMCP6, suggesting a possible location on Chromosome 1 in the E-genotype genomes.

### Functional Classification of Differentiating Genes

For functional comparison purposes, it is helpful to identify genes and other features using a controlled vocabulary. Therefore, functional classifications between the C-genotypes and E-genotypes were categorized based on the gene ontology annotation schema (GO) [28]. The Gene Ontology (GO) provides standardized terms for the description of gene products in terms of biological processes, cellular location, and molecular function [28], [29]. GO categories and individual genes having functionally significant enrichment or depletion between genomes at the species or genus level were identified using the Gene Ontologizer [30]. A detailed description of how GO terms are identified as significant is given in (Cain et al., in review) [31].

**Figure 5:**
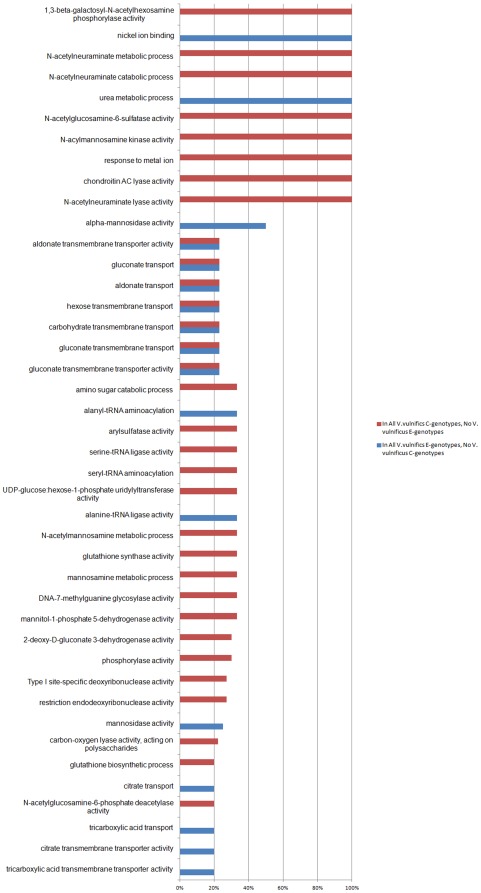
Gene Ontology (GO) functional differences between C- and E- genotypes. Figure shows GO functional categories which are enriched in C-genotypes of *V. vulnificus* relative to E-genotypes (blue) or E-genotypes relative to C-genotypes (red). Percentages represent percent of genes under each category that are differential between the genotypes. Percentages of less than 20% are not depicted.

Figure 5 summarizes differences in GO function content between the C-genotypes and E-genotypes of *V. vulnificus*. The differential functional analysis shows that GO terms mannitol-1-phosphate 5-dehydrogenase and N-acetylneuraminidase are significantly enriched in the C-types with an adjusted p-values of 2.42^E-04^ and 1.13^E-05^, respectively. Specifically, 35% of the genes associated with mannitol-1-phosphate 5-dehydrogenase activity and nearly100% of the genes with associated N-acetylneuraminidase function are found to be unique to C-types. Additionally, GO terms “chondroitin AC lyase activity” and “arylsulfatase activity” are significantly enriched with adjusted p-values of 0.0068 and 0.048, respectively. In both categories, close to 100% of the genes are only found in the C-genotype differentials. In contrast, the E-genotypes appear to be strongly enriched in genes associated with the GO functions “urea metabolic process” and “nickel ion binding”. Nearly all of the genes that fall under these GO categories are only found in the E-genotypes. Both show up as statistically significant differentials with adjusted p-values of 1.52^E-09^and 4.37^E-07^, respectively. Additionally, E-genotypes appears to have several unique genes that fall into GO categories associated with carbohydrate transport and transmembrane transporter activity for a variety of sugars and sugar derivatives.

Understanding the overall significance of these genotypic GO functional differences will require further investigation, however we propose that these differentiating functional categories may be relevant to the SPANC hypothesis which describes the balance between self-preservation and nutritional competence in bacterial genomes [32], [33].

### Chromosomal location of differential genes

It has been previously suggested that the second chromosome in the *Vibrionaceae* family may play a role in adaptation to environmental changes [34]. Our genome comparison revealed that the majority of the C-genotype differential genes (Table 2) are located on the second chromosome of each strain, with the exception of a small number of genes in *V. vulnificus* MO6-24/O (VVMO6_02633, VVMO6_02634, and VVMO6_02635). Based on the location of the E-genotype differentiating genes in the LCB alignments (Figure 2), we were able to approximate the likely chromosomal location of the E-genotype differentials in Table 3. If a gene was located inside a conserved block that appeared in Chr. 1 in the CMCP6 reference genome, we assigned it to Chr. 1 or likewise to Chr. 2 if it was found in a conserved block matching Chr. 2. This analysis suggests that E-genotype differentials such as the PKD domain-containing protein and the PTS system are found on Chr. 2, while a permease locus is most likely found on Chromosome 1. The location of differentiating genes with a urea metabolic process GO term identification can't be definitively determined based on this assembly. Our observations are consistent with increased plasticity of the second chromosome, which could potentially offer further insight into the genetic diversity found within the species, and the proposed divergence of this species into two distinct ecotypes.

### Characteristic features of the E-genotype genomes

As previously mentioned, *V. vulnificus* C- and E-genotypes have been shown to exhibit differences in pathogenicity and environmental distribution. In addition, previous examination of several housekeeping and putative virulence-associated genes has revealed a number of genetic polymorphisms suggesting that these two genotypes are in the process of diverging into distinct ecotypes [11], [27]. One hypothesis of particular interest, referred to as the SPANC (self-preservation and nutritional competence) balance, could potentially offer insight into the niche adaptation and differentiation seen in *V. vulnificus* C- and E-genotypes. The SPANC hypothesis has been well characterized in *E. coli* and demonstrates that clonal populations can experience genetic mutations and phenotypic changes as a result of physiological stress under conditions such as nutrient starvation. These changes often lead to variations in the activity of the global gene regulator, sigma factor σ^S^ (*rpoS*), which governs the general stress response. Decreased RpoS activity can lead to the development of specialized populations which are less resistant to stress but have broader nutritional capabilities and a higher affinity for low nutrient concentrations, whereas the original population is more stress tolerant but less nutritionally competent [32], [33]. In aquatic environments, in which nutrients are often limiting and competition for resources is intense, such modifications could confer a selective advantage for these bacterial strains.

It seems plausible that this trade-off between self-preservation (stress resistance) and nutritional competence could be a factor driving the diversification of *V. vulnificus* species. By completely sequencing three E-genotypes of *V. vulnificus*, we were able to examine what genes are unique to E-genotypes. As noted above, the GO functional gene content differences between C- and E-genotypes showed that the sequenced E genomes have significant enrichment for genes associated with metabolic functions such as urea and nitrogen cycle metabolism (Figure 5), suggesting that the E-genotypes may possess versatile metabolic capabilities. Previous laboratory studies support this finding demonstrating that when *V. vulnificus* C- and E-genotypes are grown in co-culture, E-genotypes are favored under nutrient rich conditions (Rosche and Oliver, unpublished). In addition, both C- and E-genotypes are enriched for GO functions associated with transmembrane transport of various organic compounds (Figure 5). Indeed, these genes appear to possess the same functionality for C- and E-genotypes, however multiple sequence alignment of the protein sequences reveals very little homology. This finding suggests that the genes associated with these GO terms have either diverged considerably between the two genotypes or are completely different genes that serve the same function. Future investigations should be performed to examine what effect these genetic differences could have on the metabolic capabilities of each genotype.

In the E-genotypes, we also identified genes associated with specific attachment proteins which would likely facilitate environmental survival. Polycystic kidney disease I domain (PKD) is a unique domain that can be found within chitinases, which has been proposed to enhance the hydrolysis of insoluble chitin [35]. Manual annotation of individual gene differentials revealed that E-genotypes have a unique PKD domain-containing protein, which is most closely related to its homolog in *Alteromonadales* TW-7, with a significant blast hit (e-value of 7.00^e-62^). Using site directed mutagenesis of conserved aromatic residues within the PKD domain, Orikoshi *et al.* [36] were able to demonstrate that the PKD domain of chitinase A in *Alteromonas* sp. strain *O-7* was required for effective binding and hydrolysis of chitin. As noted by Grimes *et al.* [37], several chitinases and putative chitinases have been identified in two previously sequenced clinical strains of *V. vulnificus* (CMCP6 and YJ016). However, the PKD gene found in the newly sequenced E strains may be a chitinase that is unique to E-genotypes. The ability to attach to chitin is important for *Vibrio* spp. as it facilitates DNA transformation, believed to be critical for horizontal gene transfer in this genus [38].

The ability to cope with the rapid and potentially stressful transition from the oyster environment to the human host likely requires a variety of stress resistance genes that provide the bacterium with protection and the ability to survive in this seemingly hostile environment. Previous studies have demonstrated the need for stress regulators to aid in survival under a variety of stressful conditions, such as starvation, osmotic stress, low pH, non-optimal temperatures, and oxidative damage [39]. Studies investigating the ability of *V. vulnificus* to survive stressful conditions have shown that C-genotypes are significantly better able to survive in complement-activated human serum than E-genotypes [40]. Rosche et al. demonstrated that C-genotypes exhibit better cross-protection when exposed to multiple stresses, such as osmotic shock followed by H_2_0_2_ exposure or elevated temperature [27]. C-genotypes appear to be physiologically more stress tolerant, and this suggest that the SPANC hypothesis may apply in *Vibrio vulnificus*, in that C-genotypes are more capable at self-preservation, while E-genotypes carry additional genes that suggest they may be more capable of nutritional competence. Sequence alignments of the *rpoS* gene for all six sequence strains did not indicate any major genetic polymorphisms, and only resulted in a few amino acid substitutions. The nucleic acid sequence is ∼99% identical and the coded protein 98.5% identical. Other genes that may affect the SPANC balance [41] are similarly well conserved. Future studies will need to be performed to investigate the roles of E- genotype specific genes under relevant conditions such as nutrient limitation in order to validate this hypothesis.

### Characteristic features of the C-genotype genomes

#### Genotype classification with Mannitol transport and fermentation genes

Mannitol transport and fermentation genes were found to be present in the C-genotype strains but not in the newly sequenced E-genotype strains. Mannitol has been correlated with virulence-associated genotypes (*vcgC* and 16S rDNA type B) [25]. This lack of a mannitol operon (consisting of a dehydrogenase, a phosphotransferase system component, and an operon repressor) in the sequenced E-type strains was identified in a previous study, and confirmed by our sequencing [42], [43]. This differentiating feature was also identified in a recent analysis of short-read sequence fragments from four other E-type strains [18]. It is important to note that while many E-genotype strains lack the mannitol operon, phenotypic and molecular testing by our laboratory has shown that 40% of 73 total tested E-type strains contain the mannitol operon and are able to ferment this sugar [42], [43]. The strains sequenced in this study and in the study by Gulig et al. [18] were among those previously known, before sequencing, to be unable to ferment mannitol, and future sequencing should include E-genotype strains that are able to ferment mannitol, to provide a more extensive comparison between these two phenotypes.

#### Genomic XII region

Cohen et al. (2007) used MLST data to identify a 33-kb genomic island (region XII) on the second chromosome of V. vulnificus [14]. This region contained an arylsulfatase gene cluster, a sulfate reduction system, two chondroitinase genes, and an oligopeptide ABC transport system, none of which were found in their “lineage II” (our E-genotype) isolates. They suggested that this region may play a role in the pathogenic process, as both arylsulfatases (see discussion below) and the chondroitin sulfate proteoglycan degrading chondrotinase have been speculated to be involved in the penetration of epithelial cells [44], [45]. The authors thus speculated that region XII, along with others, could give cells of the C-genotypes a selective advantage in their relationships with aquatic environments or human hosts, or both. Gulig et al. (2010), in their V. vulnificus sequencing study, suggested that the ability to scavenge sulfate groups could facilitate survival in the human host, where free sulfur is limited [18]. Cohen et al. (2007) identified region XII in 32 of the 37 lineage I genotypes (including reference C-genotypes, V. vulnificus CMCP6, V. vulnificus MO6-24/O and V. vulnificus YJ016) they examined, but in only 3 of the 6 lineage II strains [14]. Consistent with their findings, we identified 83.3% of this region as being present only in the C-genotypes, and not in the three E-genotypes we sequenced here.

#### Arylsulfatases

Arylsulfatases occur in virtually all organisms, and are found in high levels in the digestive glands of oysters and other mollusks [46]. These enzymes hydrolyze arylsulfate ester bonds, releasing free sulfate, which is critical for microbial growth [14]. Interestingly, arylsulfatase synthesis in enteric bacteria is regulated by norepinephrine, among other monoamine compounds, which is believed to be involved in quorum sensing in the human gut [47]. A role for arylsulfatases is suggested by the finding that, in *E. coli*, they facilitate invasion of the blood-brain barrier [32]. In a major study of the genomics of *V. vulnificus* [14], the authors found that the clinical (C-genotype) strains possess a 33 kb genomic island (“region XII”) which contains an arylsulfatase gene cluster [14]. We did not observe this gene cluster in the E-genotype strains, suggesting it may be important in the pathogenesis of the C-genotype strains, possibly by allowing survival in the human gut where free sulfur is limited [14]. In the present study, VVA1632, VVA1633, VVA1634, and VVA1635, which make up the arylsulfatase gene cluster, are among the genes differentiating C-type from E-type strains (Table 3). This difference may be one component of the reduced pathogenicity of E-genotype strains relative to C-type strains.

#### Sialic acid catabolism

Sialic acids are a family of nine-carbon sugar acids that are typically located at the terminal carbohydrate ends of mucin proteins. The most abundant sialic acid is N-acetylneuraminic acid (Neu5Ac), with all of the other sialic acids being derivatives of this compound [48]. Sialic acids are found in both eukaryotes and prokaryotes and occur on many types of cells, including epithelial cells in humans [4]. Sialic acids are commonly used as a carbon and nitrogen source for enteropathogenic bacteria, and can serve as a vital substrate for invasion and survival within the human host [49], [50]. Mucin is abundant in the mucus layer overlaying intestinal epithelial cells where *V. vulnificus* adheres and begins its infective route, and previous studies have demonstrated the importance of this gene for growth, adhesion, and survival within the jejunum and colon tissues of the mouse intestine [50]. The Nan cluster (*nanA, nanE, and nanK*) is responsible for sialic acid and catabolism has been identified in several major intestinal pathogens including *V. vulnificus* [51], [52]. The E-genotypes sequenced in this study lack the major components of the sialic acid catabolism gene cluster including *nanA*. However, recent work in our lab (Taylor and Oliver, unpublished) and others investigating the presence of *nanA* in a larger number of clinical and environmental genotypes has revealed the presence of this gene in some E-genotypes [53]. However, the *nanA g*ene is less prevalent in E-genotypes and there also appears to be some correlation between the presence of this gene and C-genotypes, highlighting the need for further investigation into the function of sialic acid catabolism as a virulence factor for *V. vulnificus*.

#### The RsbRST Operon

A hallmark method for responding and adapting to environmental fluctuations involves the use of alternate sigma factors which compete for RNA polymerase and subsequently initiate the transcription of a specific subset of genes [54]. In gram negative bacteria such as *V. vulnificus*, stressful conditions such as carbon starvation, non-permissive pH values, and hyperosmolarity will induce a stress response in which sigma S (σ^S^) competes with the housekeeping sigma factor (σ^D^) thus redirecting gene expression to respond to the stress. Gram positive bacteria, such as *Bacillus subtilis* and *Staphylococcus aureus*, use a comparable alternative sigma factor (σ^B^) which governs the “general stress response” and plays an important role in virulence in these organisms. This is accomplished by activating the transcription of over 125 genes in response to a variety of stressful conditions, including temperature shifts, ethanol, salt and acid stress, and starvation [54], [55], [56]. In *B. subtilis*, σ^B^ activation is partly regulated by a large signaling complex called the RsbRST stress module (or stressosome), and a PP2C-type phosphatase, RsbU [55].

In all three C- genotypes, we identified an operon homologous to the RsbRST stress response module, the PP2C-type phosphatase, and a downstream two-component regulatory system. With the exception of a single gene encoding *rsbR,* which we found in E64MW and JY1701, this stressosome was absent in the E-genotypes we sequenced. To our knowledge, *V. vulnificus* does not possess the σ^B^ subunit of RNA polymerase, thus the role of this signaling system in *V. vulnificus* is not clear. It has been proposed by some investigators that the function of these modules may vary considerably amongst bacteria as a result of niche expansion in which incoming signals are relayed to a diverse array of regulatory systems, such as alternative sigma factors and two-component signal transducing systems [55], [56]. Further investigation should be performed to determine if the presence of this system strongly correlates with *V. vulnificus* genotype. Additionally, elucidating the role of this system in *V. vulnificus* would be of great interest and could potentially provide insight into the mechanisms of virulence and survival in this organism.

#### Cyclic-di-GMP

Cyclic-di-GMP is an intracellular signaling molecule that acts as a second messenger for integrating environmental signals and has been demonstrated to regulate several distinct cellular processes such as motility, biofilm formation, virulence, and rugose colony morphology [57]. Cyclic di-GMP levels in the cell are controlled by the activity of diguanylate cyclases (DGCs) and phosphodiesterases (PDEs), resulting in the synthesis and degradation, respectively, of cyclic-di-GMP. DGCs are characterized by a conserved GGDEF domain, whereas PDEs contain a conserved EAL or HD-GYP domain. *Vibrio* spp. have been shown to possess a large number of these regulators indicating the importance of cyclic-di-GMP signaling in this genus in their adaptation to new environments [58]. Sequence comparisons revealed that C-genotypes possess unique GGDEF family proteins that were not present in the currently sequenced E-genotypes. Interestingly, we identified one of these GGDEF family proteins (GGDEF family protein YeaJ) located in an operon with a putative two-component response regulator and a fimbrial protein Z transcriptional regulator. In *E. coli*, *yeaJ* is one of the many GGDEF domain encoding genes that differentially mediates switching between motility and curli-fimbrial mediated adhesion [59].

In other pathogens, such as *Salmonella enterica serovar Typhimurium*, *FimZ* acts as a positive transcriptional activator of type I fimbrial expression, therefore modulating ability to attach or swim in a given environment [60]. Clegg and Hughes [60] found that an increase in *FimZ* results in a lack of motility due to the down-regulation of the *flhDC* master flagellar operon. Clegg's group has also shown that *FimZ* plays a crucial role in regulating the expression of phenotypes associated with adherence to, and invasion of, eukaryotic epithelial cells [60].

#### Type IV secretory system

Type IV secretory system gene *VirB4* (VV2_0638) was found to be present in C-genotype strains (*V. vulnificus* YJ016 and *V. vulnificus* CMCP6) but absent in the newly sequenced E-genotype isolates and *V. vulnificus* MO6-24/O. Type IV bacterial secretion systems (T4SS) are responsible for the translocation of molecules such as DNA, proteins, and toxins out of the cell and into the immediate environment or the host cell [61], [62]. This system is composed of the T-pilus and membrane-associated complex which are constructed from 12 VirB proteins, several other Vir proteins, and a coupling protein (*VirD4*) [63], [64]. Of these proteins, *VirB4* serve as energizing components as these genes are associated with ATPase functionality [63], [65]. Because this system is associated with the transfer of DNA (conjugation) and also toxins, it is also often implicated with pathogenicity. Our *V. vulnificus* E-genotype strain sequencing suggests that these T4SS components are active in infections caused by C-genotypes (*V. vulnificus* YJ016 and *V. vulnificus* CMCP6). 70% of the predicted *virB* operon sequence of the T4SS has been observed to be present in the C-genotypes (*V. vulnificus* YJ016 and *V. vulnificus* CMCP6) and not in M06-24 or the E-genotypes [66]. Sequencing of more C- and E-genotypes should be performed to investigate whether the presence of this operon displays a trend towards virulent strains in *Vibrio vulnificus*.

### Summary

In conclusion, three E-genotype strains of *Vibrio vulnificus* have been sequenced to over 99% completion. The genomes have been assembled using *ab initio* methods and contig sequences have been deposited in the NCBI Whole Genome Shotgun archive. Additional Illumina sequencing is underway with the aim of complete closure of the strain JY1305 genome. We expect that effort to provide insights into structural rearrangements among the C-genotype and E-genotype strains, but we do not expect the additional sequencing to significantly alter the findings of strain-differentiating genes reported herein. Current work in progress also includes the genomic sequencing of a larger collection of *V. vulnificus* strains, encompassing the entire genomic spectrum of pathogenicity. That data will provide additional insights into the distinct genomic differences between pathogenic and non-pathogenic strains (Baker-Austin, unpublished). The comparative analysis of C- and E- genotypes confirms previous observations of putative virulence determinants that differentiate *V. vulnificus* isolated from wounds in clinical settings from environmental strains. However, the analysis also points the way to dozens of differentiating genes specific to the E-genotypes. Some of the genes potentially fit into existing functional hypotheses such as the SPANC theory, while others are as yet functionally uncharacterized.

Although the presence or absence of a particular gene in a specific genotype provides initial targets for functional differentiation, this current sequencing effort provides the *V. vulnificus* community with a valuable reference for functional study of determinants of virulence, and facilitates the future use of high-throughput approaches to assess functional differences via study of the *V. vulnificus* transcriptome. Future studies will aim to analyze gene locations and gene neighborhoods to determine if there are genotypic differences here that could account for differences in physiology; e.g. our mannitol study revealed differences in the gene arrangement of a putative hemolysin, mannitol transporter, and mannitol fermentation operon that has been shown to correlate with clinical C-genotypes [42]. We also recognize that the presence of gene homologs (e.g. virulence-related genes) in both genotypes does not necessarily indicate equivalent function - even single base pair changes can alter protein function of a particular gene, and a detailed investigation of the impact of cross-genotype differences on protein sequences is planned as a follow-up to this study.

## Materials and Methods

### Strains, Growth Conditions, and DNA Isolation

*V. vulnificus* strain JY1305 was grown overnight in Bacto™ Heart Infusion (HI) broth (BD, New Jersey) at 30°C with vigorous shaking. Cells were pelleted by centrifugation and supernatants discarded. The cells were washed three times with phosphate buffered saline (PBS) before being resuspended to a final approximate concentration of 5×10^8^ cell/ml. The MagMax™ Total Nucleic Acid Isolation Kit (Ambion) and All Prep DNA/RNA/Protein Mini Kit (Qiagen) were used for DNA extraction. The quality and quantity of DNA was evaluated spectrophotometrically with the NanoDrop ND1000 (Thermo Scientific, Wilmington, DE). A concentration of 50 ng/µL was used for next gen sequencing.

*V. vulnificus* strains JY1701 and E64MW were grown overnight with shaking in 10 ml of ASPW. Cells were pelleted by centrifugation and resuspended in 100 µl of ice-cold PBS. DNA was extracted using DNAzol (Invitrogen) according to manufacturer instructions, followed by incubation with RNase A. Subsequently, samples were purified using a phenol/chloroform/isoamyl alcohol extraction protocol. Briefly, 40 µl of 3 M sodium acetate was added to each DNA sample, followed by 440 µl of phenol/chloroform/isoamyl alcohol. Samples were centrifuged (5 min, 13,000 rpm) and ∼400 µl of supernatant was removed and mixed with an equal volume of phenol/chloroform/isoamyl alcohol. This solution was centrifuged (5 min, 13,000 rpm) and the supernatant (∼300 µl) removed and mixed with an equal volume of 24∶1 chloroform/isoamyl alcohol. Samples were subsequently centrifuged for 5 min (13,000 rpm) and the supernatant (∼200 µl) was subjected to ethanol precipitation. The DNA pellet was re-dissolved in 50 µl 1×TE buffer and stored at −80°C. The quality and quantity of DNA was subsequently ascertained spectrophotometrically using a NanoDrop ND1000 (NanoDrop Technologies, Wilmington, DE).

### Genome sequencing and assembly

*V. vulnificus* JY1305 was sequenced at the Virginia Commonwealth University using Roche/454 Titanium technology [67]. One complete sequencing plate was used for this genome. *V. vulnificus* E64MW and JY1701 were sequenced at the BBSRC Genome Analysis Centre (Norwich, UK) also using the Roche/454 Titanium technology [67]. Quarter plates were used for both. A total of 671521, 376290, and 321096 single end reads were generated for JY1305, E64MW, and JY1701 respectively. De novo assembly with Newbler version 2.3 initially constructed 179, 269, and 269 contigs for JY1305, E64MW, and JY1701. An additional assembly was performed using the MIRA 3.2.1 de novo assembler. The default parameters for MIRA were used, except that the assembly quality parameter was changed from “normal” to “accurate”, and trace information was excluded from the assembly. MIRA constructed 159, 271, 329 contigs for JY1305, E64MW, and JY1701 respectively. Assembled contigs were compared by constructing sequence alignments using Mummer 3.0 [68]. Supplement S5 provides details of the assembly approach. A comparison of homologous contigs generated by MIRA [19] and Newbler [67] is provided in Supplement S5.

### Genome sequence comparison

Contigs from each assembly were aligned to reference genomes using the Mauve software [23]. The three E-genotype genomes, *V. vulnificus* JY1305, E64MW and JY1701, were aligned to three *V. vulnificus* C strain reference sequences [AE016795.3, CP002469.1, and BA000037.2] and longest common blocks (LCBs) were identified in each genome. *V. vulnificus* YJ016 and MO6-24/O and each set of assembled contigs for JY1305, E64MW, and JY1701 were aligned against the reference sequence *(V.vulnificus* CMCP6) to produce separate, optimal pair-wise alignments for each query sequence. The pair-wise alignments were then used to produce a multiple alignment of the newly sequenced strains, and *V. vulnificus* YJ016 and MO6-24/O, using *V. vulnificus* CMCP6, which has recently been re-annotated [22].

The LCB alignment results suggested that a plasmid sequence, present in YJ016 and thought to be present in other *V. vulnificus* strains, was absent in the newly sequenced E-strain genomes [23]. To confirm this, a PCR assay was performed on the extracted JY1305 DNA during prep, and validated the extraction of two chromosomes, and the absence of plasmid DNA. The primers used to verify chromosomal identity were *csrA* F2, *csrAR2*, *rpod* UP, *rpod* DOWN, *vvhA* F, *vvhA* R, *pepRF* F2 and *pepR3*. These were designed based on known features of the C-type genomes. The primers used to test for the presence of a YJ016-type plasmid were *vvSSF1*, *vvSSR1*, *vvF2* forward primer, and *vvR2* reverse primer. Using Primer3 [69], two sets of primers were generated for conserved regions of plasmid YJ016 and PC4602-1, with expected product length of 244 and 209 bps. The conserved sequences used for primer generation were compared to the genomic sequence of *V. vulnificus* CMCP6 and YJ016 strains using BLAST to ensure that they exclusively matched the two plasmid sequences. Primer sequences are provided in Table S3.

### Genome and gene characterization

Draft annotation of the sequences was performed using a pipeline of published microbial annotation tools. Feature determination for each strain was performed on the contig set from each sequence assembly. The feature identification methods that were used were Glimmer3.02 and GeneMark.hmm [70], [71]. Both packages are widely used feature determination applications recognized and accepted by NCBI, and both are publicly available. Glimmer3.02 was used with default parameters. An exception was that the circular chromosomes were treated as linear in the analysis. This setting was used to prevent each contig from being treated as an individual circular chromosome. GeneMark.hmm was used with default parameters. The models used for training were the two *V. vulnificus* reference organisms (CMCP6 and YJ016). Spacer sequence was added to the ends of each contig to mimic start and stop signals. The spacer sequence was 32 nucleotides in length. We used the sequence NNNNNCACACACTTAATTAATTAAGTGTGTGNNNNN, which is used at JCVI to merge contigs [http://www.jcvi.org/cms/research/projects/annotation-service/submission-guide/].

For gene identification in each of the newly sequenced strains, one of the following criteria had to be met: (1) A gene will be included in the gene list if it can be predicted by either Glimmer or GeneMark, as long as it amino acid sequence length is equal to or greater than 150. (2) A gene must be predicted by both Glimmer and GeneMark to be included in the gene list, if its amino acid sequence length less than 150. (3) A gene prediction will only be included in the gene list if it occurs in a cluster of known or hypothetical genes found in other *Vibrio* spp. The first two criteria were derived from Chen et al., 2003 [15] and were used to ensure as much consistency as possible between gene prediction methods used among all the genomes being compared. Supplement S1 describes the annotation procedure in greater detail. Supplement S1 contains the gene counts based on each criterion. When there was a conflict between a predicted gene's start position from different feature identification methods, BLASTP was used to compare the predicted gene to the sequence of its products, if available, and a start site was chosen on that basis. The target database consisted of all completely characterized bacterial genomes. ptt files. Preliminary locus tags were generated for all genes in each E-genotype genome.

tRNAScanSE was used to predicted the tRNAs in the MIRA contigs for each strain [72]. RNAHMMER was used to predict the rRNAs from the MIRA contigs for each strain [73]. In both cases, default parameter settings were used.

The reference genomes used for the comparative genomic content analysis include all the available and completely characterized *Vibrio* genomes GenBank identifiers [*Vibrio anguillarum* 775; CP002284.1, *Vibrio cholerae* LMA 3984-4; CP002555, *Vibrio cholerae* M66-2; CP001233.1, *Vibrio cholerae* MJ-1236; CP001485.1, *Vibrio cholerae O1 biovar El Tor str.* N16961; AE003852.1, *Vibrio cholerae* 0395; CP000626.1, *Vibrio fischeri* ES114; CP000020.2, *Vibrio fischeri* MJ11; CP001133.1, *Vibrio furnissii* NCTC 11218; CP002377, *Vibrio harveyi* ATCC BAA-116; CP000789.1, *Vibrio parahaemolyticus* RIMD 2210633; BA000031.2, *Vibrio sp.* Ex 25; CP001805.1, *Vibrio splendidus* LGP32; FM954973.2, *Vibrio vulnificus* CMCP6; AE016795.3, *Vibrio vulnificus* MO6-24/O; CP002469.1, and *Vibrio vulnificus* YJ016; BA000037.2].

### Gene clustering

OrthoMCL version 2.0 was used to cluster newly predicted genes with genes from other *Vibrio* spp. [74]. OrthoMCL has been shown to outperform other stand-alone methods for ortholog clustering. OrthoMCL uses an all-against-all blastp comparison of sequences as an input step followed by application of a Markov clustering procedure. The e-value cutoff for the BLASTP was 1e-5. Default parameters were used for OrthoMCL except that clusters were formed based on a shared sequence similarity of 70%, instead of the OrthoMCL default parameter value of 50%. The increase in stringency to 70% shared sequence similarity resulted in more constrained gene clusters, and reduced the chance of inappropriate clustering of partial homologs into ortholog clusters. The newly sequenced genomes were clustered first with the previously sequenced *V. vulnificus* C-type strains, and then with the 16 fully sequenced *Vibrio* species, to determine the impact of different reference sets on the orthology analysis outcome.

### Gene content comparison

The OrthoMCL clustering generated during the annotation step was used as the basis for identification of differentiating genes. Identified gene features and OrthoMCL results were stored in a locally developed OLAP data warehouse (GenoSets) that supports queries across aggregate data generated by a variety of genomic annotation and comparison methods. This system is fully described in (Cain et al. 2011, in review) [31]. Annotations for the published C-strain genomes were downloaded and parsed from the EMBL-Bank public repositories. Annotations for the novel E-strain genomes reported herein were generated as described above. Once feature boundaries were determined from the annotation and stored, gene presence-absence queries were formulated within the GenoSets system at different levels of the taxonomy hierarchy, in order to identify gene features that differentiate the three E-strains from each other, from the C-strains, and from other *Vibrio* spp.

In order to provide a standard means of comparison for feature attributes we establish relationships between features using two methods. First, we estimate orthologous relationships between genes using OrthoMCL, which uses a Markov Cluster algorithm to group putative homologs based on sequence similarity, as the primary ortholog clustering method in GenoSets. OrthoMCL has been shown to outperform other stand-alone methods for ortholog clustering [74]. For functional analysis, gene features identified in the newly sequenced *V. vulnificus* strains were associated with GO terms using homology determined through OrthoMCL clustering of BLASTP results. For functional comparison purposes, it is helpful to identify genes and other features using a controlled vocabulary. The Gene Ontology (GO) provides standardized terms for the description of gene products in terms of biological processes, cellular location, and molecular function [28], [29]. If a GO term was associated with any gene within an ortholog cluster, all genes within that cluster were also associated with that GO term. In Figure 5, we show the GO classifications, with the quantity of differentiating genes shown as a percentage of all E- and C-genotype genes.

### Phylogenetic Analysis

We identified 1748 single-copy ortholog clusters within 19 *Vibrio* spp. We performed a phylogenetic analysis following the methods used in Suzuki et al. and Hasan et al. [75], [76]. We randomly selected protein sequences of 10% of the single-copy ortholog clusters identified (175 genes) and used the sample as a basis for construction of a maximum likelihood tree, following the approach used in Hasan et al. [76]. ClustalW was used to align sequence members of each ortholog cluster independently, to minimize gene rearrangement within the multiple sequence alignment [77]. Once each individual protein alignment was built, the independent alignments were concatenated. phyML 3.0, a maximum likelihood method, was used to generate a phylogenetic species tree with 100 replicates for bootstrapping [78]. The tree was visualized with Figtree [79]. Three independent samplings were tested and all three produced trees with highly similar topologies.

## Supporting Information

Table S1 Conservation of locally collinear blocks (LCBs) in *V. vulnificus* genomes.
(DOCX)

Table S2 Summary of key gene differences between *V. vulnificus* and other *Vibrio* spp.
(DOCX)

Table S3 Primer sequences used to validate presence of genomic DNA and plasmid DNA in extracted samples.
(DOCX)

Supplement S1 Complete list of predicted genes for the draft assemblies of *V. vulnificus* JY1305, E64MW, and JY1701 and alignment position in the *V. vulnificus* CMCP6 reference genome.
(XLSX)

Supplement S2 Complete listing of LCBs (locally collinear blocks) identified by Mauve among the newly sequenced E-genotypes and the C-genotype reference strains.
(XLSX)

Supplement S3 Complete list of genes that differentiate *V. vulnificus* from other *Vibrio spp*.
(XLSX)

Supplement S4 Complete list of genes differentiating E-genotype strains from C-genotype strains.
(XLSX)

Supplement S5 Details that support the rationale for aspects of the bioinformatic analysis.
(PDF)
