# Supplementary material for: Pyrosequencing-Based Comparative Genome Analysis of Vibrio vulnificus Environmental Isolates
Source: PLoS One. 2012 May 25;7(5):e37553. doi: 10.1371/journal.pone.0037553 (PMC3360785; doi:10.1371/journal.pone.0037553)
Supplement: Table S1 — Conservation of locally collinear blocks (LCBs) in V. vulnificus genomes. (DOCX) [file pone.0037553.s001.docx]

| Criteria for LCB | # of LCBs |
| --- | --- |
| 1% (57000 Kb) or greater of the genome size | 24 |
| 90 aa or greater in length | 186 |
| Two or more C-genotypes only and length > 90aa or greater  (* C- genotype Isolate Specific) | 74 |
|  | 71* |
| Two or more E-genotypes only and length > 90aa or greater  (* E- genotype Isolate Specific) | 112 |
|  | 107* |
