# Supplementary material for: Pyrosequencing-Based Comparative Genome Analysis of Vibrio vulnificus Environmental Isolates
Source: PLoS One. 2012 May 25;7(5):e37553. doi: 10.1371/journal.pone.0037553 (PMC3360785; doi:10.1371/journal.pone.0037553)
Supplement: Table S2 — Summary of key gene differences between V. vulnificus and other Vibrio spp. (DOCX) [file pone.0037553.s002.docx]

| Strain | Locus tag | Product Description | GO ID | GO Term |
| --- | --- | --- | --- | --- |
| MO6-24/0 | VVMO6_03483 | Zinc metalloprotease | GO:0008270 | zinc ion binding+* |
|  | VVMO6_03880 | Cytolysin secretion protein | GO:0006810 | transport+* |
|  | VVMO6_03287 | RTX toxin-like Ca2+-binding protein | N/A | N/A |
|  | VVMO6_01206 | flp pilus assembly protein CpaB | N/A | N/A |
|  | VVMO6_01207 | flp pilus assembly protein, secretin CpaC | GO:0009306 | Protein secretion+* |
|  | VVMO6_01210 | flp pilus assembly protein TadA | GO:0006810 | transport+* |
|  | VVMO6_01211 | flp pilus assembly protein TadB | N/A | N/A |
|  | VVMO6_01212 | flp pilus assembly protein TadC | N/A | N/A |
| YJ016 | VVA0331 | Putative RTX toxin | N/A | N/A |
|  | VVA0537 | Zinc metalloprotease | GO:0006508 | Proteolysis +* |
|  | VV2005 | Flp pilus assembly protein TadC | N/A | N/A |
|  | VV2006 | Flp pilus assembly protein TadB | GO:0006810 | N/A |
|  | VV2007 | Flp pilus assembly protein TadA | GO:0006355 | transport+* |
|  | VV2008 | Flp pilus assembly protein CpaE-like | GO:0009306 | Regulation of transcription, DNA-dependent* |
|  | VV2010 | Flp pilus assembly protein, secretin CpaC | N/A | Protein secretion+* |
|  | VV2011 | Flp pilus assembly protein CpaB | N/A | N/A |
| CMCP6 | VV2_0032 | Zinc metalloprotease | GO:0008270 | zinc ion binding+* |
|  | VV2_0403 | Cytolysin secretion protein vvhB | GO:0044179 | hemolysis in other organism+* |
|  | VV2_1514 | RTX toxin-like Ca2+-binding protein | N/A | N/A |
|  | VV1_2330 | Flp pilus assembly protein CpaB | N/A | N/A |
|  | VV1_2331 | Flp pilus assembly protein, secretin CpaC | GO:0009306 | Protein secretion+* |
|  | VV1_2333 | Pilus assembly protein CpaE-like protein | GO:0006355 | Regulation of transcription, DNA-dependent* |
|  | VV1_2335 | Flp pilus assembly protein TadB | N/A | N/A |
|  | VV1_2336 | Type II/IV secretion system protein TadC, associated with Flp pilus assembly | N/A | N/A |
| JY1305 | VvJY1305_3952 | Cytolysin secretion protein vvhB | GO:0019835 | Cytolysis+* |
|  | VvJY1305_3640 | Zinc metalloprotease | GO:0004222 | metalloendopeptidase activity+* |
|  | VvJY1305_2030 | Flp pilus assembly protein TadB | N/A | N/A |
|  | VvJY1305_2029 | Type II/IV secretion system protein TadC,associated with Flp pilus assembly | N/A | N/A |
|  | VvJY1305_2035 | Flp pilus assembly protein CpaB | N/A | N/A |
|  | VvJY1305_2034 | Flp pilus assembly protein, secretin CpaC | GO:0009306 | Protein secretion+* |
|  | VvJY1305_2031 | Flp pilus assembly protein TadA | GO:0006810 | transport+* |
|  | VvJY1305_2032 | Flp pilus assembly protein CpaE-like | GO:0006355 | Regulation of transcription, DNA-dependent* |
| E64MW | VvE64MW_3956 | RTX toxin-like Ca2+-binding protein | N/A | N/A |
|  | VvE64MW_3058 | Cytolysin secretion protein vvhB | GO:0044179 | hemolysis in other organism+* |
|  | VvE64MW_2746 | Zinc metalloprotease | GO:0006508 | proteolysis+ |
|  | VvE64MW_2032 | Flp pilus assembly protein TadB | N/A | N/A |
|  | VvE64MW_2031 | Type II/IV secretion system protein TadC,associated with Flp pilus assembly | N/A | N/A |
|  | VvE64MW_2037 | Flp pilus assembly protein CpaB | N/A | N/A |
|  | VvE64MW_2036 | Flp pilus assembly protein, secretin CpaC | GO:0009306 | Protein secretion+* |
|  | VvE64MW_2033 | Flp pilus assembly protein TadA | GO:0006810 | transport+* |
|  | VvE64MW_2026 | FlpL (Putative flp pilus assembly protein FlpL) | N/A | N/A |
|  | VvE64MW_2034 | Flp pilus assembly protein CpaE-like | GO:0006355 | Regulation of transcription, DNA-dependent* |
| JY1701 | VvJY1701_2811 | Zinc metalloprotease | GO:0008270 | zinc ion binding+* |
|  | VvJY1701_4054 | RTX toxin-like Ca2+-binding protein | N/A | N/A |
|  | VvJY1701_3193 | Cytolysin secretion protein vvhB | GO:0006810 | Transport+* |
|  | VvJY1701_2066 | Flp pilus assembly protein CpaB | N/A | N/A |
|  | VvJY1701_2072 | Type II/IV secretion system protein TadC,associated with Flp pilus assembly | N/A | N/A |
|  | VvJY1701_2067 | Flp pilus assembly protein, secretin CpaC | GO:0009306 | Protein secretion+* |
|  | VvJY1701_2070 | Flp pilus assembly protein TadA | GO:0006810 | Transport+* |
|  | VvJY1701_2071 | Flp pilus assembly protein TadB | N/A | N/A |

|  |  |  |  |  |
| --- | --- | --- | --- | --- |
|  |  |  |  |  |
|  |  |  |  |  |
|  |  |  |  |  |

A.)

| Strains | Locus tag | Product Descriptions | GO id | GO Term |
| --- | --- | --- | --- | --- |
| *V. anguillarum* 775 | VAA_00296 | Hemolysin co-regulated protein | GO:0055114 | oxidation-reduction process* |
| *V. cholerae* LMA3894-4 | VCLMA_A1246 | Hcp | GO:0016491 | oxidoreductase activity* |
| *V. cholerae O1 biovar El Tor str*. N16961 | VC_A0017 | Hcp protein | GO:0003954 | NADH dehydrogenase activity* |
| *V. cholerae* O395 | VC0395_0116 | Hcp protein | GO:0055114 | oxidation-reduction process* |
| *V. cholerae* M66-2 | VCM66_1370 | Hcp protein | GO:0016491 | oxidoreductase activity* |
| *V. cholerae* MJ-1236 | VCD_000216 | Hcp protein | GO:0003954 | NADH dehydrogenase activity* |
| *V. fischeri* ES114 | VF_2440 | DNA-damage-inducible SOS response protein | GO:0055114 | oxidation-reduction process* |
| *V. fischeri* MJ11 | VFMJ11_2564 | DNA-damage-inducible protein F | GO:0016491 | oxidoreductase activity* |
| *V. furnisii* NCTC 11218 | Vfu_A01958 | Hcp protein | GO:0003954 | NADH dehydrogenase activity* |
| *V. harveyi* ATCC BAA-1116 | VIBHAR_05863 | Hypothetical protein | GO:0055114 | oxidation-reduction process* |
| *V. parahaemolyticus* RIMD 2210633 | VP2943 | DNA-damage-inducible protein F | GO:0016491 | oxidoreductase activity* |
| *V. sp* Ex25 | VEA_000449 | Hcp protein | GO:0003954 | NADH dehydrogenase activity* |
| *V. splendidus* LGP32 | VS_3004 | DNA-damage-inducible protein F | GO:0003954 | NADH dehydrogenase activity* |

B.)
