# Supplementary material for: Pyrosequencing-Based Comparative Genome Analysis of Vibrio vulnificus Environmental Isolates
Source: PLoS One. 2012 May 25;7(5):e37553. doi: 10.1371/journal.pone.0037553 (PMC3360785; doi:10.1371/journal.pone.0037553)
Supplement: Table S3 — Primer sequences used to validate presence of genomic DNA and plasmid DNA in extracted samples. (DOCX) [file pone.0037553.s003.docx]

| Primer Name | Primer Sequence | Identification Purpose |
| --- | --- | --- |
| csr AupF1  csr AupF2 | 5’-CGACCTTATTGCTTCCCGAT  5’-GTCAGCCTCTATCATTCAGAG | Vulnificus Chromosome 1 |
| Rpod UP  Rpod DOWN | 5'-GACCAAGCACGTACGATTC  5'-GCATTTGCATACGCTCTG | Vulnificus Chromosome 1 |
| vvhA F  vvhA R | 5'-AGCGGTGATTTCAACG  5'-GGCCGTCTTTGTTCACT | Vulnificus Chromosome 2 |
| pepRF F2  pepR3 | 5'-AGTTGTCCATATGCCTGCCTC  5'-ACGAGAGTTTCCGCTGATGA | Vulnificus Chromosome 2 |
| vvSSF1  vvSSR1 | Seq 5’ GGCAAAGCCTCTTGTAGACAC  Seq 3’ TGATAGAGTGGCAAGGGTGCC | Plasmid content |
| vvF2  vvR2 | Seq 5’ ACACACCGCATCAACGGATTGAAC (plus)  Seq 5’ GCAAGGGTGCATAAAAGGAGTGCC (minus) | Plasmid content |

Two sets of Primers were generated (the Primer 3 software) using the conserved regions of Plasmid YJ016 and PC4602-1 with expected product length of 244 and 209bps. The conserved sequenced used for primer generation were blasted against the genomic sequence of Vibrio vulnificus CMCP6 and YJ016 stains to ensure that they were exclusively for two plasmid sequences.
