# Supplementary material for: Pyrosequencing-Based Comparative Genome Analysis of Vibrio vulnificus Environmental Isolates
Source: PLoS One. 2012 May 25;7(5):e37553. doi: 10.1371/journal.pone.0037553 (PMC3360785; doi:10.1371/journal.pone.0037553)
Supplement: Supplement S5 — Details that support the rationale for aspects of the bioinformatic analysis. (PDF) [file pone.0037553.s008.pdf]

Genome Sequence Assembly and Gene Identification

Genome Sequence Assembly Statistics

The Lander-Waterman formula can be used to estimate the percentage of the genome sequence that is covered by a sequencing run of N reads, as well as to estimate the number of expected contigs [1]. For *V. vulnificus* JY1305, the initial assembly using Newbler version 2.3 resulted in approximately 33x observed coverage. The estimated coverage based on the Lander-Waterman formula was 33.3x coverage, with less than 0.1% of the genomic sequence (or approximately 5.7 Mb given the size of the genome) estimated to be found in gaps. Thus relatively little additional sequencing is likely to be needed to completely close the JY1305 genome. For the sequence data collected for *V. vulnificus* E64MW, observed coverage was 17x, and estimated coverage was 16.9x, with approximately 0.1% of the genome found in gaps. For strain JY1701, observed coverage was 13.0x we estimated coverage at 13.0x, with 0.1% of the genome found in gaps.

Genome Sequence Assembly Comparison – Newbler 2.3 and MIRA 3.0

The initial assemblies provided by the sequencing centers contained 179, 269, and 269 contigs for JY1305, E64MW and JY1701 respectively. We re-assembled the sequence reads for each strain using MIRA version 3.0 [2], which resulted in 159, 274, and 324 contigs for JY1305, E64MW, and JY1701 respectively. Tablet [3] was used to visualize the contigs and to investigate the apparent quality of both assemblies. Supplementary Figure 1, below, illustrates the difference between the Newbler 2.3 and MIRA 3.0 assemblies, showing a side-by-side comparison of the assembled sequence covering a homologous region of a large contig found in both assemblies. The difference in coverage across the region shown in this comparison is typical of the differences in assembly results from MIRA 3.0 and Newbler 2.3. A preliminary attempt at feature prediction on the initial Newbler 2.3 assemblies resulted in gene undercounts, with 24 apparent genes being missed in the JY1305 Newbler assembly, and 63 and 75 genes being missed in E64MW and JY1701 respectively. Newbler left 9263, 2897, and 2706 unassembled reads for JY1305, E64MW, and JY1701, respectively, while MIRA left 9183, 3491, and 3659 reads unassembled for JY1305, E64MW, and JY1701, respectively. Based on these observations, we chose to use the MIRA version 3.0 assembly in all subsequent analyses, contigs deposited at NCBI are from that assembly, and all results and discussion in the manuscript. Table 1 summarizes the sequence assembly statistics and the preliminary attempt at feature prediction between the two assemblies. A.) MIRA assembly statistics and B.) Newbler assembly statistics. *V. vulnificus* JY1305 had a larger depth coverage, decrease in the number of contigs produced, and from the image below the quality construction of MIRA contig is better

than Newbler contig, which is why this genome was used as the bases of all subsequently genome analysis. To allow for comparison between the genomic data of *V. vulnificus*, *V. vulnificus* E64MW and *V. vulnificus* JY1701 were ran through MIRA even though Newbler reported a high N50 value and constructed a largest contig than MIRA. However, the MIRA assembly for all three avirulent V. vulnificus genomes lead to an increase amount of gene features identified, which was previously described.

A.)

Genome	Estimated genome size	Coverage Depth	% of genome covered	# of contigs	Largest contig	N50	Feature Identification
**JY1305**	5.7 Mb	~33x	99.9%	159	489256 bp	237659 bp	2974
**E64MW**	5.7 Mb	~17x	99%	271	163962 bp	69696 bp	2977
**JY1701**	5.6 Mb	~13x	99%	329	112761 bp	36756 bp	3040

B.)

Genome	Estimated genome size	Coverage Depth	% of genome covered	# of contigs	Largest contig	N50	Feature Identification
**JY1305**	5.7 Mb	33x	99.9%	179	396819 bp	184539 bp	2950
**E64MW**	5.7 Mb	17x	99%	269	464851bp	131953 bp	2914
**JY1701**	5.6 Mb	13x	99%	269	177862 bp	64400 bp	2965

Figure 1

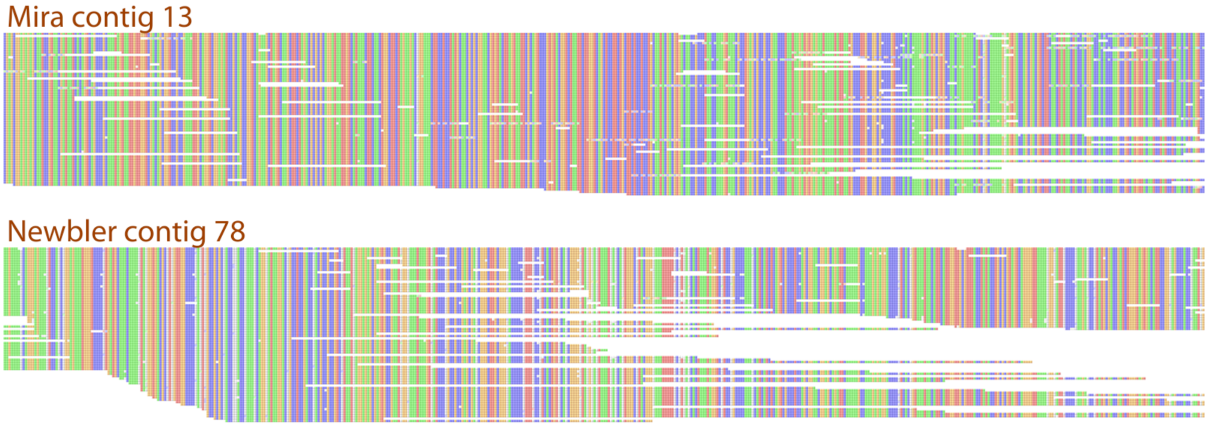

Gene prediction and functional annotation

Ab initio gene prediction using modern methods designed for prokaryotic genomes has been determined to be sufficiently accurate, usually identifying 95% or better of genes correctly [4]. NCBI recognizes three major prokaryotic genefinders for use in microbial genome annotation Glimmer [4], GeneMark.hmm [5], and Prodigal [6]. Differences in interpretation may arise when it comes to combining results from the various methods into a unified annotation. Because one of our main goals in this study was to compare the newly-sequenced E genomes to the genomes of two previously sequenced C strains, we chose to keep our analytical procedure as consistent as possible with the procedure used to develop the annotation of the reference genomes. We followed an approach used by Chen et al., 2003 [7] to establish criteria for inclusion of gene predictions from two methods, Glimmer3 and GeneMark.hmm, in the final gene lists used in our comparative analysis. Genes were included in the final gene list for each organism if they were 150 amino acids (aa) or greater in length, and were predicted by either Glimmer3, GeneMark.hmm, or both, and if they were shorter than 150 aa in length but were predicted consistently by both gene-finding approaches. In addition to these two criteria, we also included genes predicted by only one method, if they were identified as homologous to genes found in other completed *Vibrio* genomes, regardless of whether they met the length criterion. We defined homology as membership in a set of sequences that formed an unambiguous ortholog cluster with all the genomes used in this study when analyzed using OrthoMCL [8]. In exploratory analyses, we manually reviewed the annotation comparison results to determine whether the stringency of our initial criteria for gene inclusion may have caused us to miss genes that are found exclusively in the accessory genomes of the E-genotype draft genomes. When we simply applied criteria similar to Chen et al. 2003 [7] to merge the Glimmer and GeneMark.hmm annotations as described above, we left out numerous shorter genes. Accepting putative genes that were

shorter than 150 aa in length, but were supported by their membership in an ortholog cluster spanning other completely characterized *Vibrio spp.* (listed in the Material and Methods), added over 700 genes to the gene lists for each of the newly sequenced strains, as shown in the table 2, below.

Criteria for Prediction Inclusion	*V.vulnificus* JY1305	*V. vulnificus* E64MW	*V.vulnificus* JY1701
**Total Predicted**	4889	5173	5403
**Amino acid length > 150**	3482	3535	3652
**Amino acid length < 150, but predicted by both Glimmer and GeneMark**	7	8	8
**Total predicted genes following criteria from [13]**	3489	3543	3660
**Amino acid length < 150, with orthologs in other *Vibrio spp.***	746	758	765
**Total genes included in final count**	4235	4301	4425
**Predicted but not included**	654	872	978
**Percentage gene gain using orthology criterion**	21.38%	21.39%	20.90%

References

1. Lander ES, Waterman MS (1988) Genomic mapping by fingerprinting random clones: a mathematical analysis. Genomics 2: 231-239.
2. Chevreux B, Wetter T, Suhai S (1999) Genome Sequence Assembly Using Trace Signals and Additional Sequence Information. In Proceedings of German Conference on Bioinformatics. pp. 45-56.
3. Milne I, Bayer M, Cardle L, Shaw P, Stephen G, et al. (2010) Tablet--next generation sequence assembly visualization. Bioinformatics 26: 401-402.
4. Salzberg SL, Delcher AL, Kasif S, White O (1998) Microbial gene identification using interpolated Markov models. Nucleic Acids Res 26: 544-548.
5. Lukashin AV, Borodovsky M (1998) GeneMark.hmm: new solutions for gene finding. Nucleic Acids Res 26: 1107-1115.
6. Hyatt D, Chen GL, Locascio PF, Land ML, Larimer FW, et al. (2010) Prodigal: prokaryotic gene recognition and translation initiation site identification. BMC Bioinformatics 11: 119.

7. Chen CY, Wu KM, Chang YC, Chang CH, Tsai HC, et al. (2003) Comparative genome analysis of *Vibrio vulnificus*, a marine pathogen. Genome Res 13: 2577-2587.
8. Li L, Stoeckert CJ, Jr., Roos DS (2003) OrthoMCL: identification of ortholog groups for eukaryotic genomes. Genome Res 13: 2178-2189.
